# Fluorometric immunocapture assay for the specific measurement of matrix metalloproteinase-9 activity in biological samples: application to brain and plasma from rats with ischemic stroke

**DOI:** 10.1186/1756-6606-6-14

**Published:** 2013-03-23

**Authors:** Kimberly E Hawkins, Kelly M DeMars, Changjun Yang, Gary A Rosenberg, Eduardo Candelario-Jalil

**Affiliations:** 1 Department of Neuroscience, McKnight Brain Institute, University of Florida, Gainesville, FL 32610, USA; 2 Department of Neurology, University of New Mexico Health Sciences Center, Albuquerque, NM 87131, USA

**Keywords:** Matrix metalloproteinase-9 activity, Fluorescence resonance energy transfer peptide, Immunocapture assay, Focal cerebral ischemia

## Abstract

**Background:**

Matrix metalloproteinases are important factors in the molecular mechanisms leading to neuronal injury in many neurological disorders. Matrix metalloproteinase (MMP)-9 is up-regulated after cerebral ischemia and neuroinflammation and is actively involved in blood–brain barrier disruption. Current methods of measuring MMP-9 activity, such as gelatin-substrate zymography, are unspecific and arduous. Here we developed an immunocapture assay with high efficiency, specificity, and sensitivity for quantifying endogenously active as well as total MMP-9 activity.

**Results:**

A fluorescence resonance energy transfer (FRET) peptide-based immunocapture assay was developed that enables the accurate assessment of total and active forms of MMP-9 in complex biological samples. The FRET assay demonstrated correct and efficient binding of MMP-9 to a mouse monoclonal MMP-9 antibody and high specificity of the immunocapture antibody for MMP-9. Total and active levels of MMP-9 were measured in rat brain homogenates, plasma, human HT-1080 conditioned media, and RBE4 endothelial cell lysates. The FRET immunocapture assay yielded highly similar results for total MMP-9 activity when compared to gelatin-substrate zymography.

**Conclusions:**

We suggest that the new FRET peptide-based immunocapture assay is a viable replacement of zymography for sensitive and high throughput quantification of MMP-9 activity in biological samples.

## Background

Matrix metalloproteinases (MMPs) are a family of 23 members of calcium- and zinc-dependent endopeptidases with the ability to degrade extracellular matrix (ECM) proteins, including collagen, fibronectin, laminin, elastin, and proteoglycans [1-4]. The MMPs are widely studied due to their involvement in several human diseases including cancer, osteoarthritis, inflammation, neurodegeneration, and cerebrovascular diseases.

MMPs are produced as a zymogen (latent pro-MMP devoid of enzymatic activity), but upon activation, they participate in many physiological as well as pathological events. The pro-domain (pro-MMP) contains a cysteine residue that acts as a zinc-coordinating ligand masking the catalytic domain of the protease (‘cysteine switch’) and preventing the zinc atom in the catalytic domain from being available for enzymatic function [5]. Proteolytic cleavage of the pro-domain by proteases (including other MMPs) [6,7], oxidative modifications of the thiol group on the zinc-binding cysteine (e.g., S-nitrosylation) [8-10], or conformational changes due to substrate binding [11] result in the activation of the MMP.

Among the MMPs, the gelatinases (MMP-2 and MMP-9) as well as MMP-3 (stromelysin-1) and MMP-13 (collagenase-3) have been recognized as key players in the molecular mechanisms leading to neuronal injury in neurodegenerative conditions, traumatic brain injury, meningitis, multiple sclerosis, and stroke [4,12-15]. Damage to the blood–brain barrier (BBB) is a well-established neuropathological mechanism following brain injury, and activation of MMPs is critical in BBB breakdown under these conditions. Brain edema, hemorrhage, and neuronal death are the main consequences of BBB disruption following brain damage.

MMP-9 (gelatinase B) is significantly upregulated in animal models of cerebral ischemia [16-21], traumatic brain injury [22-26], and neuroinflammation [27-29]. A large body of evidence strongly implicates MMP-9 in the detrimental molecular cascades leading to BBB dysfunction in these conditions. In stroke, MMP-9 plays a principal role in hemorrhagic conversion and vasogenic edema in animal models [16,19,21,30-34] and stroke patients [35-40]. Severe neurological deficits and brain tissue loss positively correlate with elevated MMP-9 levels in stroke patients, who are at higher risk for developing life-threatening cerebral edema and hemorrhage [35,37,38,41-46].

Gelatin-substrate zymography is extensively used for the detection of gelatinase activity (MMP-2 and MMP-9) in biological samples [1]. This electrophoretic methodology is simple to perform and relatively sensitive, but may demand arduous work when a large number of samples need to be tested. A shortcoming of zymography is that it only provides a measure of total MMP-2/-9 expression (pro-MMPs plus active MMPs). Identification of active forms of these proteases in the gels is not always possible because of the low levels of expression, making net levels of activity for each gelatinase difficult to accurately quantify [47]. In addition, assessment of MMP-2/-9 expression in gelatin zymograms by quantifying active bands migrating at lower molecular weight might underestimate MMP activity since it has been shown that pro-MMPs could be activated by oxidative stress without proteolytic cleavage. This type of activation process does not appreciably change their molecular weight [9,48].

The majority of MMP-9 is in a latent form, but it is the active form that exerts biological activity. Specific peptide substrates for MMP-9, and in general for any particular MMP, are very difficult to design because most MMPs share proteolytic recognition sequences [47,49-53]. Two methods have been described to specifically detect MMP activity in complex biological samples. Both use an immunocapture antibody to first immobilize the specific MMP to a 96-well plate followed by monitoring of the proteolytic activity using chromogenic or fluorogenic peptides [49,54].

Based on these previously reported methods, here we describe the development of a high-throughput and highly sensitive MMP-9 activity assay able to detect endogenously active MMP-9 in biological samples. One of the main modifications in our assay is the initial coating of the plate with protein A/G and the use of a highly sensitive fluorescence resonance energy transfer (FRET) peptide substrate for MMP-9. By using protein A/G, which binds to the Fc region of immunoglobulins, we are able to immobilize and correctly orient the antibody for maximum MMP-9 immunocapture. This critical step in our methodology results in a more efficient and correct binding of the antibody to the plate compared to immobilization by passive absorption. The use of a commercially available FRET peptide having 5-carboxy-fluorescein (5-FAM) as a donor is another important improvement in our assay yielding a very strong fluorescent signal upon cleavage of the peptide by MMP-9. The use of other FRET pairs for measuring MMP activity have been proven to yield low fluorescence signals and consequently less sensitivity compared to 5-FAM peptides [55].

**Figure 1:**
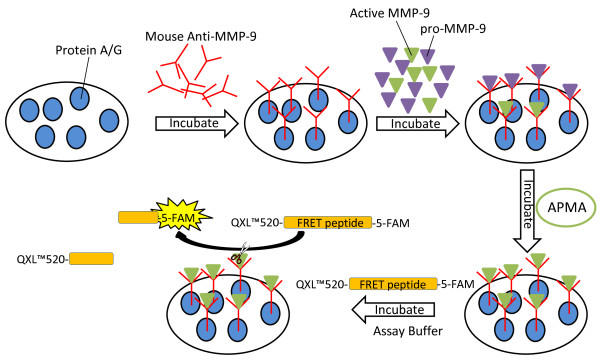
**FRET peptide-based immunocapture assay for MMP-9.** Mouse monoclonal anti-MMP-9 was added to a 96-well plate coated in Protein G to facilitate binding to the Fc region of the antibody, thus leaving the Fab region in an optimal orientation for tightly binding MMP-9. After incubation with the antibody, biological samples containing both active and pro-MMP-9 were added to the plate and allowed to incubate with the antibody to facilitate tight binding. In experiments that required activation of pro-MMP-9 to quantify total levels, APMA is added to activate the pro-MMP-9. Intact FRET peptide, in which the QXL™520 fragment is quenching the fluorescence of the 5-FAM donor, is added to the wells. Catalytically active MMP-9 is able to cleave the FRET peptide, allowing the fluorescent 5-FAM fragment to be read and monitored at excitation/emission wavelengths of 485/528 nm.

The general principle of our assay is presented in Figure 1. We validated this novel assay in complex biological matrices including rat brain homogenates, plasma, cell culture media from HT-1080 cancer cells, and RBE4 endothelial cell lysates. This sensitive fluorometric immunocapture assay specific for MMP-9 could be widely utilized to quantify active MMP-9 in biological samples and should be considered as a replacement for gelatin-based zymography. By changing the immunocapture antibody and FRET peptide, the activity of other members of the MMP family could be measured.

## Results and discussion

### Rat and human MMP-9 specifically and efficiently binds to mouse monoclonal anti-MMP-9

**Figure 2:**
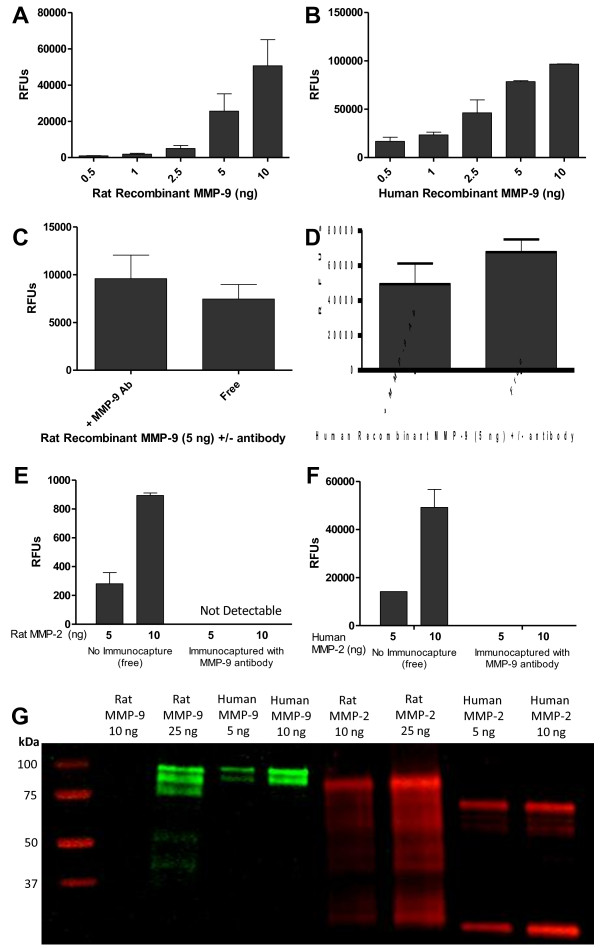
**Rat and human MMP-9 specifically and efficiently binds to mouse monoclonal anti-MMP-9.** Recombinant MMP-9 activity and specificity was measured. (**A**) Dose response of rat and (**B**) human recombinant MMP-9 was determined (n = 3–5). (**C**) Activity of free versus immunocaptured rat and (**D**) human MMP-9. Free MMP-9 was not bound to the MMP-9 antibody but instead was free-floating. Rat and human free vs. immunocaptured MMP-9 activity did not differ (*p* > 0.05, n = 4). All relative fluorescent units were normalized to a substrate control with 0 ng MMP-9. (**E**) Rat and (**F**) human recombinant MMP-2 was incubated with or without antibody to check the specificity of the MMP-9 antibody. No signal was detected with rat MMP-2 and very little signal was detected with human MMP-2 in the immunocapturing condition whereas free active MMP-2 did have fluorescent activity (n = 2–3). (**G**) Immunoblot for anti-MMP-9 specificity to MMP-9. Rat and human recombinant MMP-9 (92 kDa) and MMP-2 (72 kDa) were probed with the mouse monoclonal anti-MMP-9 then detected with a secondary antibody against mouse (in green). To confirm presence of MMP-2, an anti-MMP-2 antibody was used and counterstained (in red). 10 ng of rat MMP-9 was not enough protein to have significant binding with the MMP-9 antibody.

We first needed to determine the efficacy of the immunocapture antibody. The dose response of the mouse monoclonal anti-MMP-9 to rat and human recombinant MMP-9 (0.5, 1.0, 2.5, 5.0, and 10 ng) was examined (Figure 2A and 2B). Both rat and human MMP-9 fluorescent activity (measured in relative fluorescent units, or RFUs) increased as the quantity of recombinant MMP-9 increased. Figures 2C and 2D demonstrate the activity of “free” (no antibody coating on the plate, MMP-9 is added directly to the plate) versus immunocaptured rat and human MMP-9 (5 ng). No significant binding interference with the activity or activation of the gelatinase was observed with rat or human recombinant MMP-9. These findings indicate that binding of MMP-9 to the antibody is highly efficient as binding does not significantly reduce the catalytic ability of MMP-9.

A significant advantage in our methodology is to coat the black 96-well plate with protein G or use commercially available protein G coated plates. A more efficient immobilization of the anti-MMP-9 antibody to the plate results in a better sensitivity as shown in the Additional file 1: Figure S1. We compared the RFUs values for three different concentrations of active human recombinant MMP-9 between plates that were initially coated with protein G and non-coated plates. In the non-coated plates, the anti-MMP-9 antibody was immobilized by passive absorption to Fluotrac 600 high-binding plates overnight at 4°C. As presented in the Additional file 1: Figure S1, MMP-9 activity was significantly higher (p < 0.01) when the immunocapture antibody was immobilized to protein G-coated plates compared to plates in which the antibody was immobilized by passive absorption.

To study the specificity of the antibody for MMP-9, we assessed cross-reactivity with MMP-2. Because MMP-9 and MMP-2 have structural similarities, it was possible that MMP-2 binding and activation could contribute to the observed fluorescence. When recombinant rat MMP-2 was immunocaptured with the MMP-9 antibody, no detectable activity was observed (Figure 2E). This indicates rat MMP-2 is not recognized and captured by the MMP-9 antibody and is likely removed during a washing step. Recombinant human MMP-2 was immunocaptured at barely detectable activity levels, which indicates that human MMP-2, at very high doses, either will bind to the anti-MMP-9 or can bind to the protein G on the plate. However, the binding of human MMP-2 is probably not competitive with MMP-9 binding in biological samples. Rat and human MMP-2, pre-activated with APMA, was added to a plate with no antibody and combined with the FRET peptide. The free-floating MMP-2 (5 and 10 ng) was then able to interact with the FRET peptide to produce fluorescence (Figure 2E and 2F). This indicates that the lack of fluorescence when MMP-2 was immunocaptured with the mouse anti-MMP-9 is not due to lack of activity of the recombinant MMP-2. Specificity of the MMP-9 antibody for rat and human MMP-9 but not for rat or human MMP-2 was further demonstrated via immunoblotting (Figure 2G). Rat and human MMP-9 and MMP-2 were loaded in the gels. Initially, only MMP-9 was probed for using the mouse anti-MMP-9 antibody (shown in green). No bands were detected in the MMP-2 lanes, indicating that monoclonal MMP-9 antibody does not recognize rat or human MMP-2. To confirm the presence of MMP-2 in the gels, a second probe using an anti-MMP-2 antibody was performed (shown in red). These data indicate the monoclonal mouse antibody is highly specific for MMP-9.

### Total and endogenously active levels of MMP-9 are measurable with the FRET peptide-based immunocapture assay in multiple biological samples

**Figure 3:**
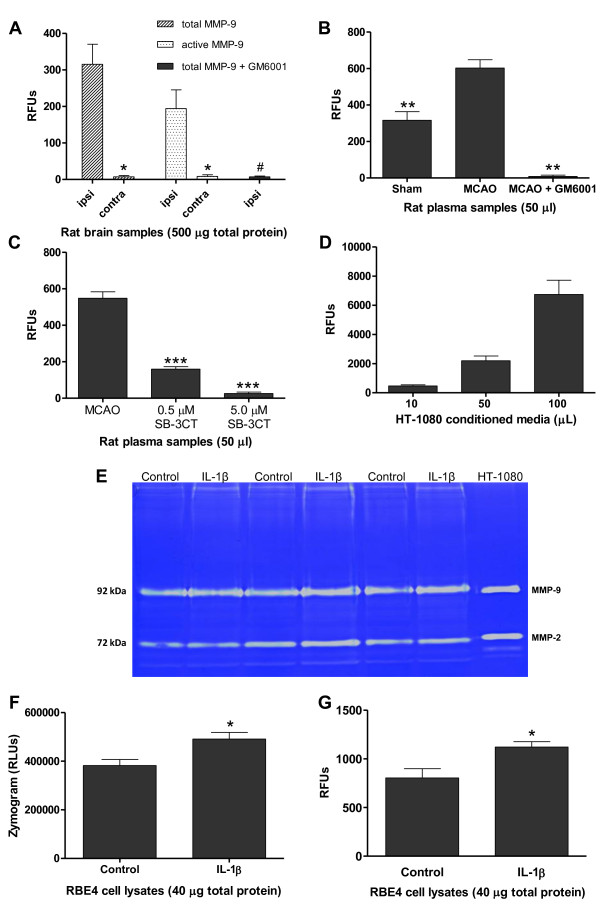
**Total and endogenously active MMP-9 are measurable with the FRET peptide-based immunocapture assay in multiple biological samples.** Brain homogenates and plasma obtained from rats that underwent MCAO and sacrificed 48 h later were used in the new FRET peptide assay. (**A**) Total and active MMP-9 activity was measured in 500 μg total protein from ipsilateral and contralateral cerebral cortex. GM6001 (10 μM) was added to total MMP-9 ipsilateral samples. Total ipsilateral, active ipsilateral, and total ipsilateral + GM6001 compared by ANOVA with Newman-Keuls post-hoc test. * Statistically significant decreases compared to ipsilateral, *p* < 0.001, n = 7–8. # Statistically significant decrease compared to total ipsilateral, *p* < 0.01, n = 4–8. (**B**) Total MMP-9 activity recorded from 50 μL plasma from sham and MCAO rats. GM6001 (10 μM) added to MCAO plasma samples significantly reduced MMP-9 activity. Compared by ANOVA with Newman-Keuls post-hoc test. ** Statistically significant difference compared to MCAO, *p* < 0.001, n = 5. (**C**) Total MMP-9 activity from MCAO rat plasma samples (50 μL) and MCAO with the gelatinase inhibitor SB-3CT (0.5 μM and 5.0 μM). MMP-9 activity was significantly reduced with addition of SB-3CT in a dose-dependent manner. Compared by ANOVA with Newman-Keuls post-hoc test. *** Significant decrease compared to MCAO, *p* < 0.0001, n = 5. (**D**) Total MMP-9 activity in 10, 50, and 100 μL HT-1080 conditioned media (n = 4–6). (**E**) Zymogram, (**F**) densitometric analysis, and (**G**) FRET assay of total MMP-9 activity in lysates from RBE4 cells treated with IL-1β (10 ng/mL) compared to controls. Treatment with IL-1β significantly increased total MMP-9 levels in RBE4 cells as seen with zymography (Student’s *t*-test, * *p* < 0.05, n = 3) and the FRET assay (Student’s *t*-test, * *p* < 0.05, n = 3 independent experiments).

For this assay to be a viable technology in many research laboratories, it must be able to detect MMP-9 levels in many biological samples. We first demonstrated the ability of the assay to detect both total and endogenously active levels of MMP-9 in homogenates of rat cerebral cortex and plasma obtained from animals subjected to transient focal cerebral ischemia by middle cerebral artery occlusion (MCAO). Total and endogenously active levels of MMP-9 were recorded in ipsilateral (same hemisphere as ischemia) and contralateral (opposite hemisphere from ischemia) samples with 500 μg total protein (Figure 3A). Ipsilateral samples had more activity compared to contralateral samples for both total and active MMP-9. The addition of a classic matrix metalloproteinase inhibitor, GM6001 (total concentration 10 μM), to ipsilateral samples greatly reduced the activity of MMP-9. We have successfully detected total and endogenously active levels of MMP-9 in rat brain homogenates using as low as 50 μg total protein which emphasizes the high sensitivity of the assay (data not shown).

Figure 3B shows MMP-9 activity in 50 μL of rat plasma samples from sham-operated rats (surgery but no occlusion of the middle cerebral artery) and MCAO rats. Plasma from the sham rats had significantly less total MMP-9 activity than MCAO rats. Again, with the addition of GM6001 (10 μM) to the MCAO rat plasma, there was a large reduction in the activity of MMP-9. We also used the gelatinase inhibitor SB-3CT (0.5 μM and 5.0 μM) to check the specificity of the inhibition of MMP-9 in the plasma samples, as SB-3CT has very high selectivity to MMP-2 and MMP-9 compared to GM6001 [56,57]. When SB-3CT was added to the MCAO plasma samples, there was a significant decrease in MMP-9 activity dependent on the concentration of SB-3CT added (Figure 3C).

This assay can also detect levels of human MMP-9 in conditioned cell culture media. As Figure 3D illustrates, increasing amounts of conditioned media (10, 50, and 100 μL) from the human fibrosarcoma cell line HT-1080, when immunocaptured with the mouse anti-MMP-9, coincides with greater MMP-9 activity.

Rat brain endothelial 4 (RBE4) cells are known to respond to inflammatory stimuli such as IL-1β [58]. We tested whether levels of MMP-9 can also be ascertained in cell lysates. Lysates were taken from RBE4 cells that were subjected to 24 h of stimulation with interleukin-1β (IL-1β; 10 ng/mL). When compared to control cell lysates, levels of MMP-9 in lysates from treated cells were significantly increased as seen by gelatin-substrate zymography (Figure 3E and 3F). When measured with the new FRET peptide assay, IL-1β-treated cell lysates had significantly more MMP-9 activity than controls (Figure 3G), indicating that this new assay demonstrates a similar trend in the MMP-9 levels in RBE4 cells after treatment with IL-1β as zymography.

Following ischemia/reperfusion, levels of MMP-9 are dramatically increased in the brain, specifically the ipsilateral hemisphere [32,40]. Our FRET peptide-based assay reflects this well-known biological response and is sensitive enough to measure only endogenously active MMP-9 in samples, unlike zymography which primarily measures total levels of MMP-9. MMP-9 that is activated by oxidative stress is more easily quantified in this assay method as well, since activating pro-MMP-9 in this way does not substantially alter the molecular weight of MMP-9, therefore making it difficult to distinguish from the pro-MMP-9 in a zymogram. Inhibition of MMP-9 proteolytic activity by adding GM6001 to brain tissue, and GM6001 as well as SB-3CT in rat plasma, greatly reduced the emitted fluorescence, indicating it is MMP-9 cleaving the FRET peptide and not a different protease. That this assay also works in cultured cell media and cell lysates indicates it is not limited in sample medium as it can assess MMP-9 levels in multiple biological samples.

### MMP-9 activity correlates in gelatin-substrate zymography and FRET peptide-based immunocapture assay

**Figure 4:**
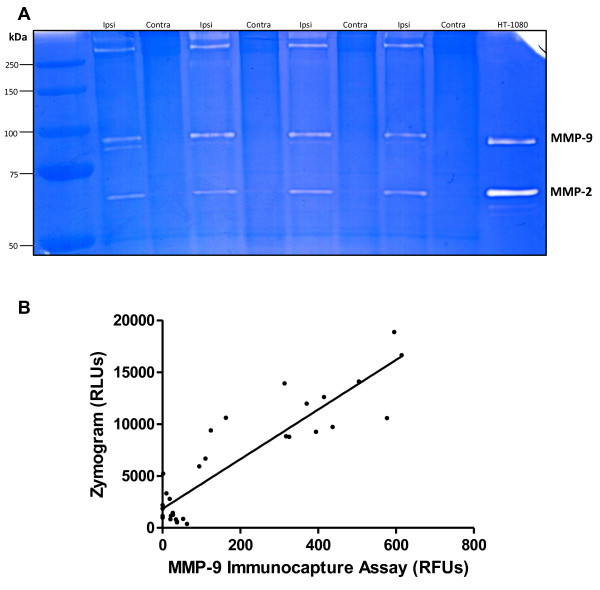
**MMP-9 activity correlates in gelatin-substrate zymography and FRET peptide-based immunocapture assay.** Results of MMP-9 activity from rat MCAO brain tissue homogenate samples were compared between zymography and the FRET immunocapture assay. (**A**) Representative zymogram of 50 μg total protein from rat cerebral cortex homogenates, ipsilateral (ipsi) and contralateral (contra) hemispheres, and HT-1080 conditioned media. MMP-9 (94 and 88 kDa) and MMP-2 (72 kDa) are shown. Quantification of MMP-9 included the 94 and 88 kDa bands. (**B**) Correlation of relative lysis units (RLUs) quantified from zymography and relative fluorescent units (RFUs) recorded from the FRET peptide-based assay from 16 rat ipsilateral and 16 rat contralateral cerebral cortex samples. Compared with linear regression analysis, R^2^ = 0.8024, *p* < 0.0001, n = 32.

Gelatin-substrate zymography is a popular and accepted method for measuring levels of MMP-2/-9 in samples. However, if large amounts of samples need to be tested, repeated zymography can be very time-consuming. For all of this effort, only total levels of MMP-2/-9 can be accurately detected rather than both active and total levels of the gelatinases. Our proposed FRET peptide-based immunocapture assay provides the ability for many samples to be tested at once while also allowing for a choice of total or endogenously active levels of MMP-9 to be quantified. For this assay to be a viable replacement of gelatin-substrate zymography, relative fluorescence units (RFUs) observed for a sample in the immunocapture assay must be similar to relative lysis units (RLUs) recorded from gelatin zymography. To check for this correlation, total MMP-9 levels were studied from 16 rat MCAO cerebral cortex homogenates (ipsilateral and contralateral) in both the immunocapture assay and gelatin-substrate zymography. A sample zymogram of rat cortex samples and HT-1080 conditioned media indicating both gelatinases is shown in Figure 4A. As seen in the FRET peptide assay (Figure 3A), the zymogram data illustrates MMP-9 activity in ipsilateral samples as greater than contralateral samples. To determine whether the data from each method are comparable, we plotted the RLUs from the zymograms against the RFUs obtained from the FRET assay (Figure 4B). Total MMP-9 activity levels highly correlate between the two methods, indicating MMP-9 activity reported from zymography is very similar to that determined from the FRET peptide-based assay. These data indicate that the FRET-based immunocapture assay is a possible quantitative replacement of gelatin zymography for measurement of MMP-9.

Another advantage of the FRET-based assay is the flexibility offered. Other MMPs can easily be measured by altering the specific immunocapture antibody and/or the FRET peptide used. While gelatin zymography is limited to measurement of only total MMP-2 and MMP-9 activity, the FRET peptide assay can quantify total and active levels of the gelatinases.

## Conclusion

Gelatin-substrate zymography is the go-to method for measurement of gelatinase activity in samples despite lengthy experiment times and restrictions on observable data. A new method, the FRET peptide-based immunocapture assay, has increased throughput, flexibility, and sensitivity and is proposed here as a viable quantitative replacement of zymography.

## Materials and methods

### Materials

The protein G coated 96-well plates and the mouse monoclonal anti-MMP-9 antibody were purchased from Thermo Fisher Scientific (Cat. Nos. 15157 and MS-817-P, respectively; Rockford, IL, USA). The FRET peptide was purchased from AnaSpec (Cat. No. 60570–01; San Jose, CA, USA). 4-aminophenylmercuric acetate (APMA), GM6001, and SB-3CT were purchased from Sigma-Aldrich (Cat. Nos. A9563-5G, M5939, and S1326, respectively; St. Louis, MO, USA).

Rat and human recombinant MMP-9 were purchased from R&D Systems (Cat. Nos. 5427-MM and 911-MP-010; Minneapolis, MN, USA). Rat and human recombinant MMP-2 were purchased from Abcam (Cat. Nos. ab39304 and ab40964; Cambridge, MA, USA). Crude brain tissue homogenates were prepared from rats with right side focal ischemia. Unfixed frozen cortex samples from both the ipsilateral and contralateral hemispheres were weighed and homogenized in 1% sodium dodecyl sulfate (SDS) buffer containing 150 mM NaCl, 50 mM Tris–HCl pH 7.6, 1% IGEPAL® CA-630, and 1% sodium deoxycholate. Just before the buffer was added to the brain samples, HALT Protease Inhibitor Cocktail (Cat. No. 78430; Thermo Fisher Scientific), HALT Phosphatase Inhibitor cocktail (Cat. No. 78428; Thermo Fisher Scientific) and 0.5 M EDTA were added at 10 μL/mL of homogenization buffer. Chilled tissue was homogenized in the buffer for 15-20 s using a Tissue-Tearor homogenizer (Cat. No. 985370; BioSpec, Inc, Bartlesville, OK, USA) then sonicated twice using a Vibra-Cell™ sonicator (Model VCX130PB; Sonics & Materials, Inc, Newtown, CT, USA). Resulting tissue homogenates were centrifuged at 14,000 x*g* for 20 min at 4°C in an Eppendorf microcentrifuge Model 5430R, and the supernatants aliquoted and stored at −80°C until used.

### Rat stroke model and sample preparation

Focal cerebral ischemia was induced by temporary middle cerebral artery occlusion (MCAO) in male Wistar rats (280–320 g; Harlan Laboratories, Indianapolis, IN, USA) using the intraluminal filament method as described previously by our group [32,59]. Briefly, rats were anesthetized with isoflurane in medical-grade oxygen and a midline vertical incision was made in the neck to expose the common carotid artery (CCA), external carotid artery (ECA) and internal carotid artery (ICA). The CCA was ligated permanently with a 4–0 silk suture and a vascular clip was temporarily placed in the pterygopalatine artery to prevent incorrect insertion of the occluding filament. A loose tie was placed over the ICA and ECA bifurcation with 4–0 silk suture and vascular clips were placed in the ICA and ECA. A small arteriotomy was made in the CCA approximately 2 mm proximal to the carotid bifurcation. A 4–0 silicone-coated filament (Cat. No. 403523PK10; Doccol Corporation, Sharon, MA, USA) was inserted through the CCA and advanced 18–20 mm inside the ICA until a mild resistance was felt. The occluding filament was left in place for 90 min and animals were allowed to recover from anesthesia. Eight to ten minutes before the end of the occlusion period, animals were re-anesthetized with isoflurane inhalant anesthesia, and the filament was gently retracted to allow reperfusion of the MCA territory.

After 48 h of reperfusion, animals were deeply anesthetized with pentobarbital (150 mg/kg; i.p.) and a blood sample was withdrawn from the vena cava into a heparinized syringe. Blood (1.5 mL) was quickly mixed with 50 μL of heparin (1000 U/mL) and centrifuged for 10 min at 2,000 x*g* to obtain the plasma. Rats were perfused intracardially with ice-cold saline and brains were harvested and dissected into ipsilateral (stroke side) and contralateral cerebral cortex and striatum. Samples were immediately frozen on dry ice and stored at −80°C until use.

### Cell culture

HT-1080 human fibrosarcoma cells were obtained from American Type Culture Collection (ATCC, Manassas, VA, USA) and maintained in DMEM:F12 medium (Life Technologies, Carlsbad, CA, USA) supplemented with 10% fetal bovine serum (FBS; Cat. No. 10082–147; Life Technologies), 100 U/mL penicillin and 100 μg/mL streptomycin in a humidified incubator at 37°C and 5% CO_2_. At 80-85% confluency, cells were washed with Dulbecco’s PBS and fresh media without FBS was added. After 24 h, cell culture media was collected and spun down at 5,000 x*g* for 10 min at 4°C. Aliquots of the HT-1080 conditioned media were prepared and stored at −80°C until use.

Rat brain endothelial (RBE4) cells were cultured in alpha-MEM/Ham’s F-10 Nutrient (1:1 solution; Cat. Nos. 12571–063 and 11550–043; GIBCO, Life Technologies) supplemented with 10% heat-inactivated fetal bovine serum (Cat. No. F4135; Sigma), 1% penicillin/streptomycin (Cat. No. 15140–122; GIBCO, Life Technologies), and 1% Geneticin (300 μg/mL; Cat. No. ALX-380-013-G001; Enzo Life Sciences). RBE4 cells were seeded in rat tail collagen I (50 μg/mL; Cat. No. C3867; Sigma) coated 6-well plates (20,000-30,000 cells/cm^2^) and maintained at 37°C, 5% CO_2_ incubator for 2 days before treatment. When cells reached 80-90% confluency, IL-1β (10 ng/mL; Cat. No. 501-RL/CF; R&D Systems, Inc., Minneapolis, MN, USA) was added to wells as treated groups. After 24 hours incubation, untreated and treated cells were washed once with ice-cold phosphate-buffered saline (PBS), and then lysed in radioimmunoprecipitation (RIPA) buffer consisting of 50 mM Tris–HCl (pH 7.6), 150 mM NaCl, 1% NP-40, 1% Sodium deoxycholate and 1% SDS plus complete protease and phosphatase inhibitor cocktails (Cat. No. 78430 and 78428; Thermo Scientific). Finally, the lysates were spun down at 14,000 xg for 15 min at 4°C. Aliquots of the supernatants were saved and stored at −80°C until use.

### Immunocapture assay and fluorometric measurement of MMP-9 enzymatic activity

The activity of MMP-9 in rat stroke brain samples was measured fluorometrically using 5-FAM/QXL™520 FRET peptide (Cat. No. 60570; AnaSpec, San Jose, CA). In the intact FRET peptide (QXL™520-Pro-Leu-Gly-Cys[Me]-His-Ala-D-Arg-Lys[5-FAM]-NH_2_), the fluorescence of 5-FAM (5-carboxy-fluorescein) is quenched by the proximity of QXL™520. Upon cleavage into two fragments by MMP-9, the fluorescence of 5-FAM can be read and monitored at excitation/emission wavelengths of 485/528 nm.

Samples were prepared in 100 μL of TCNB buffer (50 mM Tris, 10 mM CaCl_2_, 150 mM NaCl, 0.05% Brij® L23). Pierce protein G coated 96-well plates were washed three times with 200 μL TCNB buffer. Mouse monoclonal anti-MMP-9 (1 μg) in 100 μL of TCNB buffer was added to the wells and incubated for two hours at room temperature in a microplate mixer (USA Scientific, Ocala, FL, USA). Plates were then washed three times with 200 μL TCNB buffer and 100 μL of the samples were added to the wells in duplicate and allowed to incubate overnight at 4°C in a microplate mixer. After washing the plates three times with TCNB buffer, 1 mM APMA in 100 μL assay buffer (50 mM Tris–HCl pH 7.6, 200 mM NaCl, 5 mM CaCl_2_, 20 μM ZnCl, and 0.05% Brij® L23) was added to the wells to activate pro-MMP-9 in the samples to detect total levels of MMP-9, then covered in aluminum foil and incubated for 90 min at 37°C. To detect only endogenously active levels of MMP-9, APMA was not added and only 100 μL assay buffer was added to the wells. Next, 100 μL of assay buffer with 2 μM FRET peptide was added to the wells to bring total FRET peptide concentration to 1 μM. Fluorescence (relative fluorescence units, RFUs) was measured after 24 h of incubation at 37°C at 485 nm excitation and 528 nm emission in a Synergy HT Multi-mode microplate fluorescence reader (BioTek, Winooski, VT, USA) running Gen5™ data analysis software. A substrate control well was used to subtract baseline fluorescence from the sample wells.

### Fluorometric MMP-2/-9 activity measurement without immunocapture

Recombinant MMPs were activated by adding APMA for a final concentration of 1 mM and incubated for 90 min at 37°C. Active MMPs were prepared in 100 μL assay buffer and mixed with 2 μM FRET peptide in 100 μL assay buffer for a final FRET peptide concentration of 1 μM. Fluorescence was monitored after a 24 h incubation period at 37°C at the same wavelengths mentioned above.

### Gelatin-substrate zymography

Substrate-specific zymography for determination of activity of MMP-2 and MMP-9 was done on brain homogenates, HT-1080 conditioned media, and RBE4 cell lysates as described before [27,60]. Brain homogenates or RBE4 cell lysates (50 μg total protein) were mixed in 10 μL zymogram sample buffer (Cat. No. 161–0764; Bio-Rad, Hercules, CA). 15 μL of HT-1080 conditioned media at 1:10 dilution in zymogram sample buffer was loaded in the gels. Proteins were separated by electrophoresis in a SDS-PAGE gel (8%) containing 0.1% (w/v) gelatin (Cat. No. G-2500; Sigma-Aldrich) at 150 V constant voltage. Gels were then washed twice in 2.5% Triton X-100 to remove SDS for 20 min then incubated for 24 h at 37°C in incubation buffer (50 mM Tris–HCl pH 7.6 containing 200 mM NaCl, 10 mM CaCl_2_, 0.02% Brij® L23, 0.02% NaN_3_). Gels were stained with Coomassie Brilliant Blue R solution (Cat. No. B6529; Sigma-Aldrich) for 1 h then destained in 10% acetic acid for one day before being scanned with an HP Scanjet 8300 scanner. Densitometric analysis of lytic zones at 94 and 88 kDa was performed using ImageJ (freely provided by NIH). The rat MMP-9 comprises two bands running in the zymography gels at 94 kDa (glycosylated form) and 88 kDa (intermediate form) [61].

### Immunoblotting

Recombinant MMP-2 and MMP-9 were mixed in Laemmli sample buffer (Cat. No. 161–0737; Bio-Rad; Temecula, CA) containing 5% 2-mercaptoethanol and boiled for 10 min. Samples were loaded into TGX 4-20% precast gels (Cat. No. 456–1095; Bio-Rad) and subjected to electrophoresis. Proteins were transferred to Immobilon-FL polyvinylidene fluoride (PVDF) membranes using a Trans-Blot Turbo apparatus (Bio-Rad) at 25V for 30 min. Membranes were blocked for 1 h at room temperature with 5% non-fat milk in Tris-buffered saline (TBS). Membranes were incubated overnight at 4°C with primary antibodies against MMP-9 (mouse anti-MMP-9; Cat. No. MS-817-P; Thermo Scientific) and MMP-2 (rabbit anti-MMP-2; Cat. No. RPCA-MMP2; EnCor Biotechnology Inc., Gainesville, FL, USA) diluted 1:1000 in 5% milk in TBST. After washing with TBST (four times for 5 min each), membranes were incubated for 1 h at room temperature with goat anti-mouse IRDye 800CW secondary antibody (Cat. No. 926–32210; Li-Cor Biotechnology) diluted 1:30,000, and goat anti-rabbit 680RD antibody (Cat. No. 926–68071) diluted 1:40,000 in 5% milk in TBST. Protein bands were visualized using an Odyssey infrared scanner (Li-Cor Biosciences, Lincoln, NE, USA).

### Statistical methods

GraphPad Prism 5 was used for statistical analysis. Statistical significance was assessed by ANOVA followed by Newman-Keuls corrections for multiple comparisons or with a Student’s *t*-test. Linear regression analysis was used to determine correlation information and significance. Bar graphs are shown as mean ± SEM. Probability *p* < 0.05 was considered statistically significant.

## Abbreviations

MMP: Matrix metalloproteinase; ECM: Extracellular matrix; BBB: Blood–brain barrier; FRET: Fluorescence resonance energy transfer; 5-FAM: 5-carboxy-fluorescein; APMA: 4-aminophenylmercuric acetate; MCAO: Middle cerebral artery occlusion; RFU: Relative fluorescence unit; RBE4: Rat brain endothelial 4; IL-1β: Interleukin-1β; RLU: Relative lysis unit; CCA: Common carotid artery; ECA: External carotid artery; ICA: Internal carotid artery; PVDF: Polyvinylidene fluoride; TBS: Tris-buffered saline.

## Competing interests

The authors declare that they have no competing interests.

## Authors’ contributions

KEH, GAR, and ECJ conceived and designed research; ECJ led the project; KEH, KMD, CY, and ECJ performed experiments; and KEH and ECJ performed data analysis and wrote the paper. All authors read and approved the final manuscript.

## Supplementary Material

Additional file 1: Figure S1 MMP-9 activity comparison between protein G plates and non-coated plates. This experiment was conducted exactly as described in Materials and Methods with the only exception being the way the mouse anti-MMP-9 antibody was immobilized to the plate. Protein G coated plates were obtained from Thermo Fisher Scientific, and the anti-MMP-9 antibody (Cat No. MS-817-P, Thermo Fisher Scientific) was immobilized to these plates by incubating at room temperature for 2 h in a microplate mixer (see Materials and methods). In the plates that were not pre-coated with protein G, the anti-MMP-9 antibody was immobilized by passive absorption to Fluotrac 600 high-binding plates (Greiner Bio-One) overnight at 4°C (1 μg of antibody in 100 μL of phosphate-buffered saline, pH 7.4, per well). Active human recombinant MMP-9 (0.5, 5 and 10 ng) was added to the plates and incubated overnight at 4°C. The fluorescence was measured at 24 h after adding the FRET peptide to the plates (Excitation = 485 nm; Emission = 528 nm). RFUs: relative fluorescence units. **p < 0.01 and ***p < 0.001 with respect to protein G coated plates (Student’s *t*-test; n = 3 per concentration of MMP-9). Click here for file
