# Supplementary material for: Fluorometric immunocapture assay for the specific measurement of matrix metalloproteinase-9 activity in biological samples: application to brain and plasma from rats with ischemic stroke
Source: Mol Brain. 2013 Mar 23;6:14. doi: 10.1186/1756-6606-6-14 (PMC3620676; doi:10.1186/1756-6606-6-14)
Supplement: Additional file 1: Figure S1 — MMP-9 activity comparison between protein G plates and non-coated plates. This experiment was conducted exactly as described in Materials and Methods with the only exception being the way the mouse anti-MMP-9 antibody was immobilized to the plate. Protein G coated plates were obtained from Thermo Fisher Scientific, and the anti-MMP-9 antibody (Cat No. MS-817-P, Thermo Fisher Scientific) was immobilized to these plates by incubating at room temperature for 2 h in a microplate mixer (see Materials and methods). In the plates that were not pre-coated with protein G, the anti-MMP-9 antibody was immobilized by passive absorption to Fluotrac 600 high-binding plates (Greiner Bio-One) overnight at 4°C (1 μg of antibody in 100 μL of phosphate-buffered saline, pH 7.4, per well). Active human recombinant MMP-9 (0.5, 5 and 10 ng) was added to the plates and incubated overnight at 4°C. The fluorescence was measured at 24 h after adding the FRET peptide to the plates (Excitation = 485 nm; Emission = 528 nm). RFUs: relative fluorescence units. **p < 0.01 and ***p < 0.001 with respect to protein G coated plates (Student’s t-test; n = 3 per concentration of MMP-9). [file 1756-6606-6-14-S1.pptx]

## Slide 1
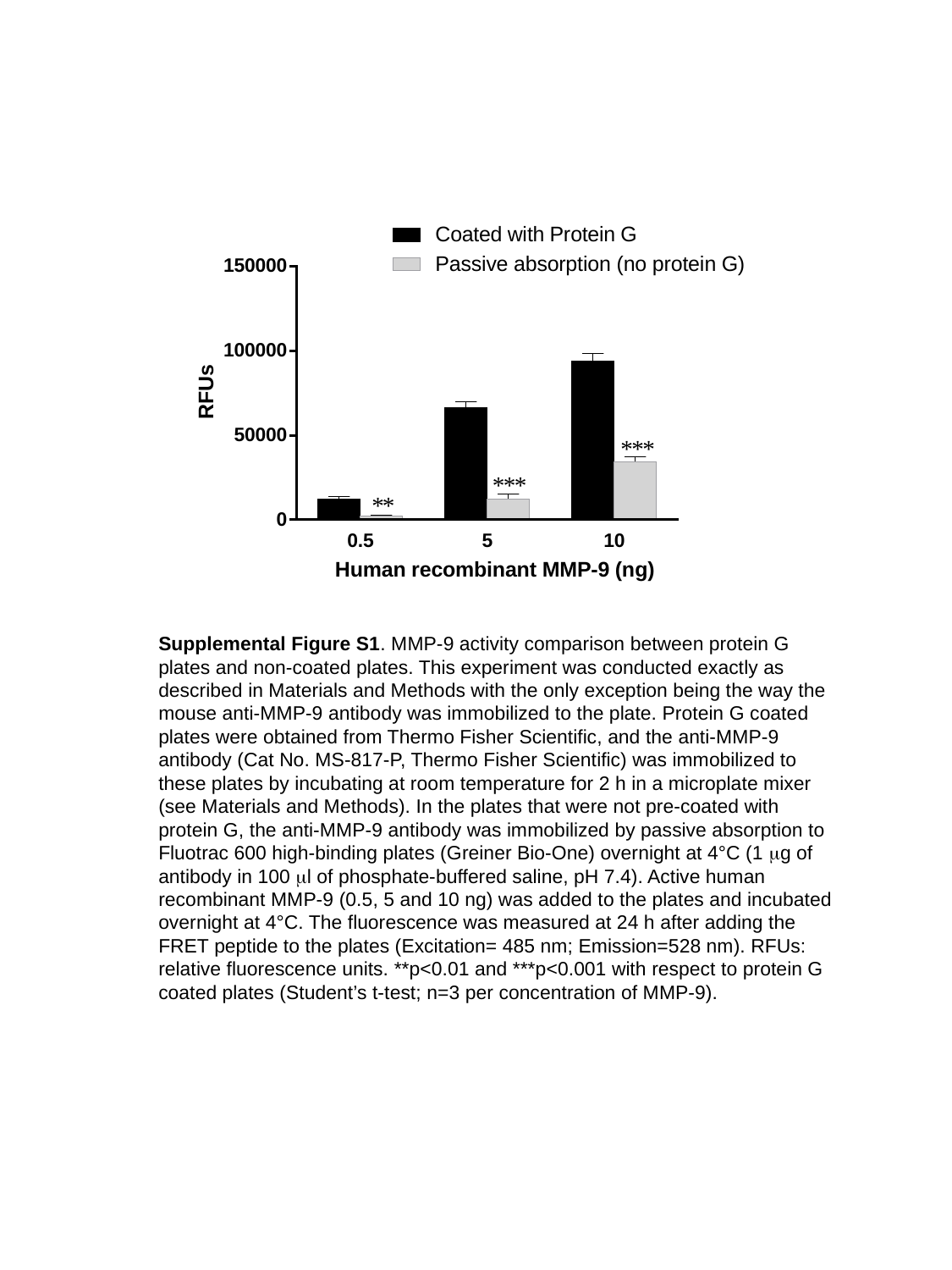

Supplemental Figure S1. MMP-9 activity comparison between protein G plates and non-coated plates. This experiment was conducted exactly as described in Materials and Methods with the only exception being the way the mouse anti-MMP-9 antibody was immobilized to the plate. Protein G coated plates were obtained from Thermo Fisher Scientific, and the anti-MMP-9 antibody (Cat No. MS-817-P, Thermo Fisher Scientific) was immobilized to these plates by incubating at room temperature for 2 h in a microplate mixer (see Materials and Methods). In the plates that were not pre-coated with protein G, the anti-MMP-9 antibody was immobilized by passive absorption to Fluotrac 600 high-binding plates (Greiner Bio-One) overnight at 4°C (1 mg of antibody in 100 ml of phosphate-buffered saline, pH 7.4). Active human recombinant MMP-9 (0.5, 5 and 10 ng) was added to the plates and incubated overnight at 4°C. The fluorescence was measured at 24 h after adding the FRET peptide to the plates (Excitation= 485 nm; Emission=528 nm). RFUs: relative fluorescence units. **p<0.01 and ***p<0.001 with respect to protein G coated plates (Student’s t-test; n=3 per concentration of MMP-9).
